# Prospective Multicenter IDE Study of the Next-Generation Precision Aspiration Thrombectomy System for Intermediate-Risk Pulmonary Embolism: The SYMPHONY-PE Trial

**DOI:** 10.1161/CIRCINTERVENTIONS.125.015815

**Published:** 2025-09-17

**Authors:** Sripal Bangalore, R. Dana Tomalty, Herman Kado, Sameh Sayfo, Adam Raskin, Arman Qamar, Andres Vargas Estrada, Kirema Garcia-Reyes, H. Gabriel Lipshutz, Srinivas Yallapragada, Sabah Butty, Sagar Gandhi, David Dexter, Justin Trivax, Farhan Ali, Michael Knox, Christopher Ramos, Youssef Al-Saghir, Vivian Bishay

**Affiliations:** Division of Cardiology, NYU Langone Health, New York (S. Bangalore).; Radiology of Huntsville, Huntsville Hospital, AL (R.D.T.).; Department of Cardiovascular Medicine, Corewell Health William Beaumont Hospital, Royal Oak, MI (H.K., J.T.).; Department of Cardiovascular Medicine, Baylor Scott and White, The Heart Hospital, Plano, TX (S.S.).; Division of Endovascular Cardiovascular Medicine, Mercy Health, Inc, Cincinnati, OH (A.R.).; Division of Interventional Cardiology and Vascular Medicine, Endeavor Health (NorthShore University HealthSystem), Glenbrook Hospital, Glenview, IL (A.Q.).; Department of Cardiology, Tallahassee Memorial Hospital, Tallahassee Research Institute, Inc, FL (A.V.E.).; Department of Diagnostic, Molecular, and Interventional Radiology, Mount Sinai Health System, New York (K.G.-R., V.B.).; Division of Interventional Radiology, Cedars-Sinai Medical Center, Los Angeles, CA (H.G.L.).; Department of Cardiology, Medical City Dallas, TX (S.Y.).; Department of Radiology, Indiana University, Indianapolis (S. Butty).; Division of Vascular Surgery, Department of Surgery, Prisma Health, Greenville, SC (S.G.).; Department of Vascular Surgery, Sentara, Norfolk, VA (D.D.).; Department of Cardiology, Medical City Fort Worth, TX (F.A.).; Advanced Radiology Services, Interventional Radiology, Corewell Health West, Grand Rapids, MI (M.K.).; Division of Vascular Surgery and Endovascular Therapy, Emory University, Atlanta, GA (C.R.).; First Coast Cardiovascular Institute, Jacksonville, FL (Y.A.-S.).

**Keywords:** anticoagulants, hemorrhage, myocardial infarction, pulmonary embolism, thrombectomy

## Abstract

**BACKGROUND:**

Mechanical thrombectomy offers a promising alternative to thrombolytic-based approaches for reducing thrombus burden and right heart strain in acute pulmonary embolism. This pivotal Food and Drug Administration–approval trial evaluated the safety and efficacy of the Symphony Thrombectomy System (Imperative Care, Inc, Campbell, CA) in acute intermediate-risk pulmonary embolism.

**METHODS:**

Patients with intermediate-risk pulmonary embolism (systolic blood pressure ≥90 mm Hg; right ventricle-to-left ventricle ratio >0.9) were enrolled without roll-ins. The primary safety end point was the rate of major adverse events within 48 hours, defined as a composite of all-cause major bleeding, device-related mortality, and serious device-related events, adjudicated by an academic independent safety board. The primary efficacy end point was the core laboratory–assessed mean change in right ventricle-to-left ventricle ratio from baseline to 48 hours. Prespecified performance goals were set for both. Exploratory end points included immediate postprocedure change in mean pulmonary artery pressure and change in modified Miller index at 48 hours. Safety was assessed in the intention-to-treat cohort and efficacy in the modified intention-to-treat cohort, excluding patients receiving nonstudy device treatments.

**RESULTS:**

Between December 2023 and May 2025, 109 patients were treated at 17 US centers (intention-to-treat), with 106 in the modified intention-to-treat cohort. The major adverse events rate was 0.9% (1/109), with an upper 97.5% CI of 5.7%, meeting the <15% safety goal (*P*<0.001). No device-related clinical deterioration, pulmonary vascular injury, or cardiac injury occurred at 48 hours; no mortality occurred through 30 days. The mean right ventricle-to-left ventricle ratio decrease was 0.44, with a lower 97.5% CI of 0.36, exceeding the >0.20 performance goal (*P*<0.001). Mean pulmonary artery pressure decreased from 29.1±7.2 to 22.2±6.6 mm Hg (24%) and the modified Miller index from 24.2±4.1 to 14.9±5.4 (38%), both *P*<0.001.

**CONCLUSIONS:**

These data support the safety and effectiveness of the Symphony Thrombectomy System in patients with acute intermediate-risk pulmonary embolism.

**REGISTRATION:**

URL: https://www.clinicaltrials.gov; Unique identifier: NCT06062329.

**WHAT IS KNOWN**

- Current treatment guidelines recommend therapy with anticoagulation alone for acute intermediate-risk pulmonary embolism though the risk of short-term morbidity and mortality remains high.
- Mechanical thrombectomy has the potential to rapidly remove thrombus and improve hemodynamics, thereby reducing the risk of short-term morbidity and mortality.

**WHAT THE STUDY ADDS**

- The Symphony Thrombectomy System is the first aspiration thrombectomy system for pulmonary embolism that combines large-bore (16F and 24F) aspiration catheters with continuous vacuum and allows for mechanical clot engagement with the ProHelix.
- No device-related major adverse events occurred at 48 hours combined with no mortality through 30 days underscore the favorable safety profile and potential clinical impact.
- Significant improvements in right ventricle-to-left ventricle ratio, pulmonary artery pressures, and clot burden, with a short median device time (31 minutes) across 17 US sites, may inform clinical decision-making.

Pulmonary embolism (PE) is a life-threatening cardiovascular emergency and remains the third most common cause of cardiovascular death, following myocardial infarction and stroke.^1,2^ While anticoagulation remains the cornerstone of therapy and is a guideline-recommended therapy, it does not permit active and rapid removal of thrombus and may be insufficient in patients with hemodynamic compromise or progressive right heart strain.^3^ Systemic thrombolysis can reduce mortality in high-risk PE but is associated with a significant risk of major bleeding, including intracranial hemorrhage.^4^ In response, catheter-directed therapies have emerged as a means of achieving rapid thrombus debulking while potentially minimizing systemic exposure to thrombolytics. However, catheter-directed therapy still relies on low-dose thrombolysis.^5,6^ Furthermore, slow and incomplete thrombus removal, distal embolization, and risk of bleeding remain ongoing concerns.^7^

The Symphony Thrombectomy System is a novel, next-generation continuous aspiration system with full vacuum control developed for the treatment of venous thromboembolism and PE. The system was designed to address limitations and workflow inefficiencies associated with manual aspiration–based approaches. Unlike earlier generation aspiration devices, the Symphony Thrombectomy System incorporates a continuous vacuum generator that can be controlled using the device handle, which allows a single operator to simultaneously control deep pulse vacuum and catheter advancement, potentially reducing procedural variability and improving workflow. The system also includes multiple large-bore catheter sizes (24F and 16F) to support efficient thrombus removal in both the main pulmonary arteries and the smaller segmental arteries.

Initially, the Symphony Thrombectomy System was intended for use in the peripheral vasculature and held 510(k) clearance in the United States for nonsurgical removal of fresh, soft emboli and thrombi, as well as for injection, infusion, or aspiration of contrast media and other fluids into or from a blood vessel. The SYMPHONY-PE (Symphony Pulmonary Embolism) study aimed to support an expanded indication for thrombus removal from the pulmonary arteries in patients with acute PE. The study was approved by the US Food and Drug Administration (FDA) under an IDE (Investigational Device Exemption) and included prespecified performance goals, informed by prior trials in similar populations, for the primary safety and efficacy end points.

## Methods

This report was prepared in accordance with the Consolidated Standard of Reporting Trials for interventional, nonrandomized studies.^8^ The data supporting the findings of this study are available from the corresponding author upon reasonable request.

### Study Device Description

**Figure 1.**
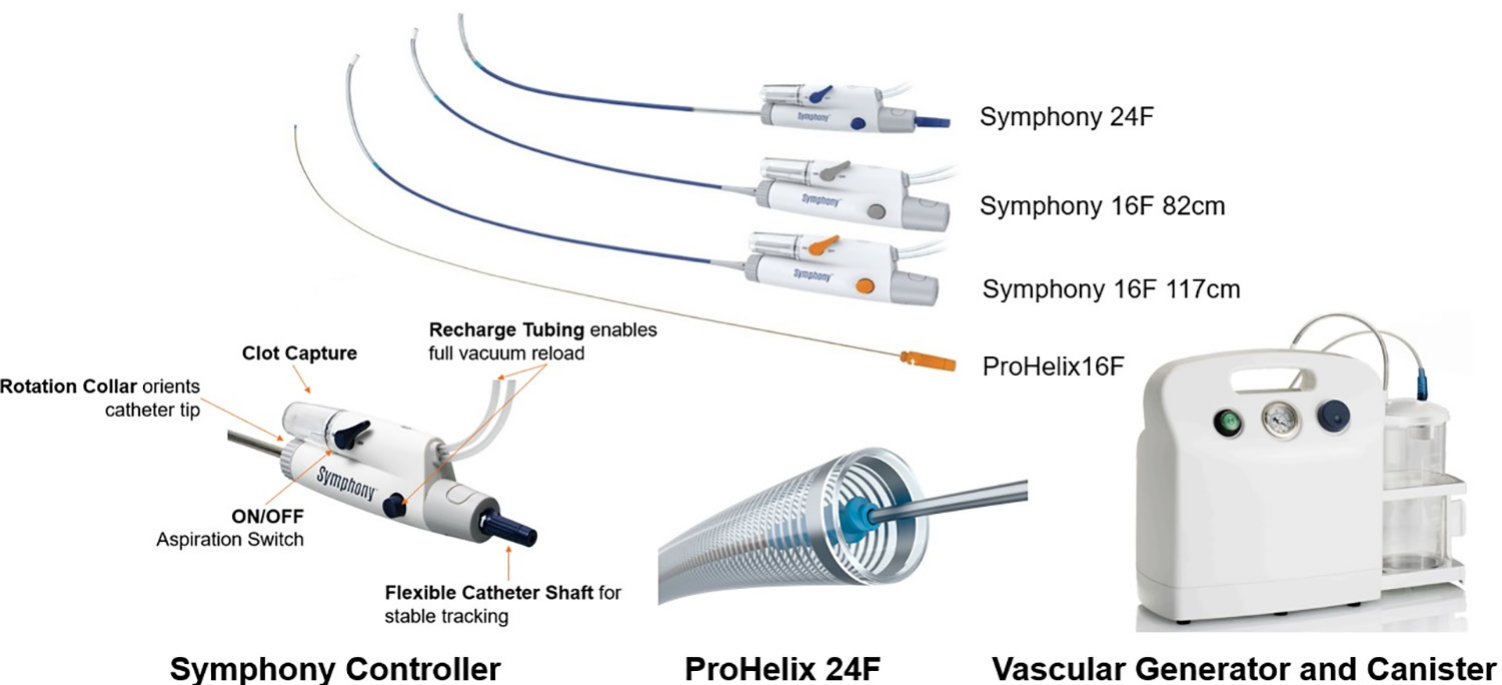
**Symphony thrombectomy system.** The system includes a generator pump unit that provides on-demand vacuum and 2 available catheter sizes (16F and 24F) designed to facilitate effective thrombus removal during the procedure. The 16F catheter can be telescoped through the 24F catheter. This figure is provided by the study sponsor and is published with permission.

The Symphony Thrombectomy System includes the following components: 24F and 16F Symphony catheters; matching 24F and 16F dilators; the 24F Symphony Advance Long Dilator; 24F and 16F Symphony ProHelix; and the generator, canister, and tube set (Figure 1). The catheters deliver continuous vacuum from the Generator directly to the thrombus, while the ProHelix may be used to assist with clot engagement and removal if needed. The catheters feature a lubricious hydrophilic coating along the 40-cm (24F) and 55-cm (16F) distal end of the shaft. Aspiration is performed by connecting the catheter to the generator via the tube set and the canister. When needed, the Symphony ProHelix is introduced through the catheter over a guidewire and remains within the catheter during the procedure. The ProHelix is manually rotated via a proximal handle, which causes the distal tip to rotate and assist in dislodging and removing thrombus during aspiration if needed.

### Study Design and Oversight

This pivotal IDE trial was a prospective, multicenter, single-arm interventional study designed to evaluate the safety and efficacy of the Symphony Thrombectomy System for the treatment of patients with acute, intermediate-risk PE. The study protocol was developed by the first and last authors, who served as national co-principal investigators, in collaboration with the sponsor. The protocol was approved by the FDA and the institutional review boards of all participating sites before subject enrollment. There were no amendments to the protocol during the course of the study. The trial was prospectively registered on https://www.clinicaltrials.gov (NCT06062329) before enrollment of the first subject and was conducted in compliance with applicable FDA IDE regulations, International Organization for Standardization 14155, Good Clinical Practice guidelines, and the principles of the Declaration of Helsinki. All analyses were prespecified and conducted according to the approved statistical analysis plan. The authors conducted a critical review of the manuscript and affirmed the accuracy and completeness of the data. No confidentiality agreements were in place that restricted the publication of the trial results.

### Patient Population

Inclusion and exclusion criteria were designed to ensure that the study population had baseline characteristics comparable to those in the prior studies of mechanical thrombectomy. Key inclusion criteria were age between 18 and 80 years, systolic blood pressure ≥90 mm Hg, and evidence of right ventricular (RV) dilation, defined as an RV-to-left ventricle (LV) ratio >0.9 based on the investigator’s assessment. Key exclusion criteria included thrombolytic use within 14 days of baseline imaging, kidney dysfunction (serum creatinine >1.8 mg/dL or estimated glomerular filtration rate <45 mL/min), hematocrit <28% or hemoglobin <9 g/dL, and pulmonary hypertension with a peak pulmonary artery pressure (PAP) >70 mm Hg as measured by right heart catheterization. Full eligibility criteria are provided in Table S1.

A patient was considered enrolled once the patient provided written, study-specific informed consent, and the Symphony thrombectomy system was introduced into the body. Roll-in patients were not permitted in the trial, and no single site was allowed to enroll >20% of the total evaluable subjects.

### Trial Procedures and Follow-Up

Following the baseline assessment that included computed tomography pulmonary angiogram, eligible patients underwent aspiration thrombectomy with the Symphony Thrombectomy System. Patient preparation, sedation, vascular access, procedural anticoagulation, and vascular closure were performed per the local standard of care. PAP was measured before starting the thrombectomy and after completing the thrombectomy.

Following vascular access and placement of the introducer sheath, the prepared Symphony Thrombectomy System, comprising the aspiration catheter and dilator, was introduced over a guidewire and advanced until the tip of the dilator reached the desired location in the target vessel. The choice of aspiration catheters (24F, 16F, or a telescoping configuration with the 16F advanced through the 24F) was left to the discretion of the operator. Once the catheter tip was positioned at the thrombus, the dilator was withdrawn while maintaining the position of both the catheter and the guidewire. The tube set was then connected to the catheter handle and the continuous vacuum source (canister/generator). Pulse aspiration was initiated by toggling the on/off switch. The catheter was then maneuvered to all target locations using the dilator for support as needed. To remove the clot from the Symphony clot container, the operator pressed and held the vent button, rotated the clot container in the unlocked position, detached it from the handle, and emptied the thrombus.

The Symphony Thrombectomy System was mandated as the first and only device used for thrombectomy. Use of other thrombectomy devices was considered a protocol deviation and permitted only when medically necessary.

Follow-up assessments were conducted at 48 hours post-treatment (−8/+12 hours), at discharge, and at 30 days (±7 days) post-procedure (Table S2). At the 48-hour follow-up, computed tomography pulmonary angiogram was performed to assess the RV/LV ratio and residual thrombus burden, and was sent to the core laboratory for independent, centralized evaluation. Until discharge, all subjects were monitored for signs of clinical deterioration and adverse events. At the 30-day follow-up, any ongoing or new adverse events were reviewed.

### Study End Points and Definitions

#### Primary Safety Performance Goal End Point

The primary safety performance goal end point was the rate of major adverse events (MAEs), a composite of all-cause major bleeding, device-related death, and device-related serious adverse events (defined as clinical deterioration, pulmonary vascular injury, or cardiac injury) within 48 hours of the procedure. The academic independent safety board adjudicated all potential MAE and device-related safety events per the prespecified definitions provided in Table S3.

#### Primary Efficacy Performance Goal End Point

The primary efficacy performance goal end point was the mean decrease in RV/LV between baseline and 48 hours post-procedure, as assessed by the core laboratory using baseline and postprocedure computed tomography angiogram.

#### Secondary Safety End Points

Secondary safety end points included all-cause major bleeding, device-related mortality, device-related clinical deterioration, device-related pulmonary vascular injury, and device-related cardiac injury at 48 hours. Additional secondary end points included PE-related mortality, all-cause mortality, device-related SAEs, and symptomatic PE recurrence at 30-day follow-up.

#### Additional Prespecified Exploratory End Points

Additional prespecified exploratory end points included change in modified Miller index between baseline and 48 hours post-procedure, estimated aspirated blood volume (blood+saline in canister), and use of thrombolytics or nonstudy devices within 48 hours. Mean and systolic PAPs were also prospectively measured and summarized along with procedure time metrics.

### Statistical Analysis

Unless otherwise noted, normally distributed continuous data are presented as mean±SD and nonnormally distributed data as median (interquartile range [IQR]). Categorical data are presented as frequency counts and percentages, with corresponding 95% CIs for end points. A Wilson score CI was used for the primary safety end point, and Clopper-Pearson exact intervals were used for all other binary end points. A paired *t* test was used to evaluate the primary efficacy end point and changes in PAP.

**Figure 2.**
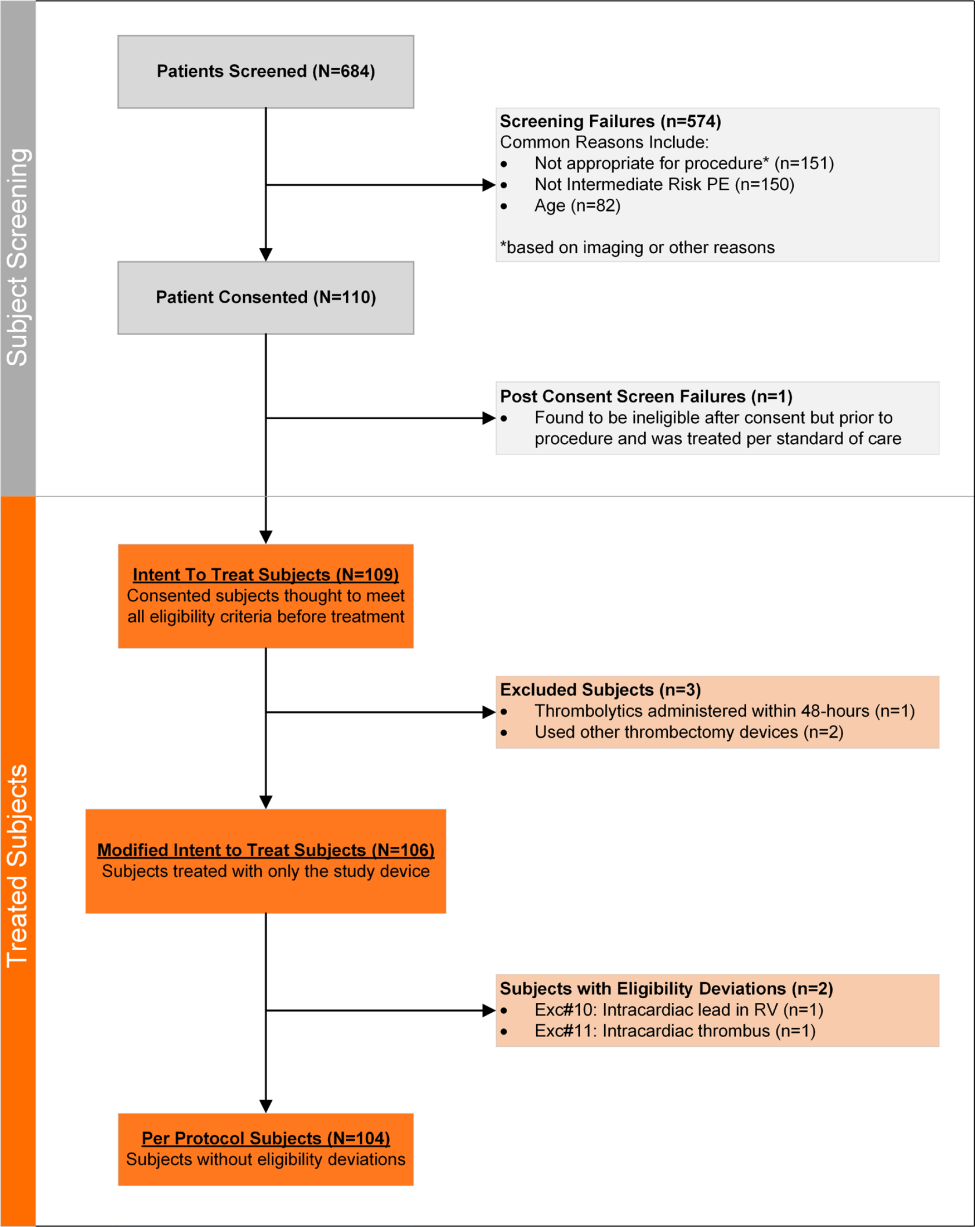
**Trial screening and enrollment.** Flow of enrolled subjects from screening through study completion, including exclusions and reasons for withdrawal. PE indicates pulmonary embolism; and RV, right ventricle.

All safety analyses were conducted on the intent-to-treat (ITT) population, comprising subjects who underwent aspiration thrombectomy with the study device (Figure 2). The primary efficacy and additional exploratory analyses were conducted on the modified ITT population, excluding subjects who received adjunctive therapy within 48 hours post-procedure, consistent with other PE IDE trials and FDA guidance. All other prespecified end points were also evaluated in the modified intention-to-treat population. Sensitivity analyses were completed using mean imputation and tipping point methods to determine whether missing data may have impacted study conclusions. The per protocol cohort, a subset of the modified intention-to-treat cohort that excludes subjects with eligibility criteria deviations (Figure 2), was also evaluated to determine whether these deviations had an impact on study results.

Performance goals were prespecified for the primary safety and primary efficacy end points with the intent of demonstrating better safety and efficacy than observed in prior PE studies for thrombolytic and anticoagulation-based treatment strategies.^9–18^ The performance goal for the primary safety end point was for the upper bound of the 1-sided 97.5% CI on the MAE rate to be <15.0%, the highest major bleeding rate reported in the literature when treating PE with thrombolytics. The performance goal for the primary efficacy end point was for the lower bound of the 1-sided 97.5% CI on the mean reduction in RV/LV to be >0.20, the largest reduction observed in the reviewed literature when treating PE with anticoagulation alone.

The Symphony Thrombectomy System was expected to achieve a comparable mean reduction in the RV/LV ratio and rate of MAE to other FDA-approved mechanical thrombectomy devices available when the study was designed. In prior studies evaluating these devices (FLARE [FlowTriever Pulmonary Embolectomy Clinical Study],^19^ EXTRACT-PE [A Prospective, Multicenter Trial to Evaluate the Safety and Efficacy of the Indigo Aspiration System in Acute Pulmonary Embolism],^20^ and FLASH [The FlowTriever All-Comer Registry for Patient Safety and Hemodynamics]^21^), the weighted average RV/LV reduction was 0.38 (range, 0.36–0.43), and the MAE rate, using the same definition as SYMPHONY-PE, was 1.3% (range, 1.0%–1.7%). An initial sample size of 84 evaluable subjects provided ≥99% for each primary end point and >98% overall study power. The target was increased to 100 subjects to detect rare but serious adverse events and to align with sample sizes in similar pivotal studies.

All statistical analyses were performed using R statistical software, version 4.5.0 (R Foundation for Statistical Computing, Vienna, Austria).

## Results

Between December 2023 and May 2025, 17 of the 19 activated sites in the United States enrolled at least 1 subject. In total, 109 patients were enrolled and treated with the Symphony Thrombectomy System (Figure 2; ITT cohort). Two patients received additional treatment with nonstudy devices, and one patient received systemic thrombolytic therapy within 48 hours of the procedure and was excluded from the modified ITT cohort (N=106 patients).

### Baseline Characteristics

Among the 109 patients, the median age was 62 (IQR, 51–69) years, and 39.4% of patients were female (Table 1). Nearly half of the patients (48.6%) had systemic hypertension, and recent surgery was reported in 19.3% of patients. Prior deep vein thrombosis (DVT) was reported in 37.6% of patients, and at the time of presentation, 63.3% of patients had concomitant DVT. The median time from presentation to the index procedure was 16.7 (IQR, 5.5–28.6) hours. Most patients (95.4%) had bilateral or saddle thrombus with only 4.6% of patients having unilateral thrombus burden (Table S4). Baseline laboratory findings met the study eligibility criteria and showed elevated biomarkers consistent with intermediate-high–risk PE (Table S5).

**Table 1.**
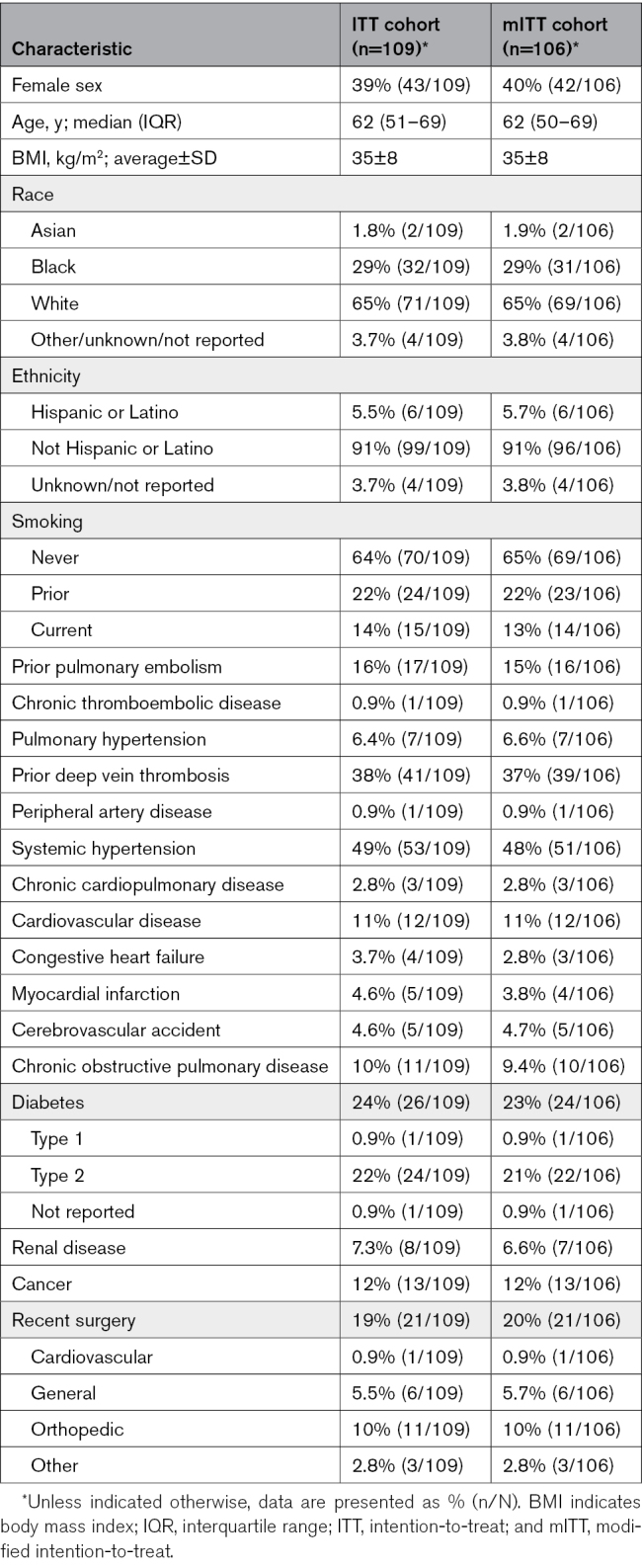
Baseline Characteristics and Comorbidities of Study Patients

### Procedural Characteristics

The majority of patients (88.9%) were treated under conscious sedation, 2.8% under general anesthesia, and 8.3% with no sedation (Table S6). The median device use time was 31 (IQR, 20–53) minutes, and the total procedure time was 61 (IQR, 41–85) minutes. ProHelix was used to assist aspiration in 11% (12/109) of patients, and aspirated clot morphology was reported as acute in 61.5% (67/109) of patients.

The mean PAP decreased significantly from 29.1±7.2 mm Hg pre-aspiration to 22.2±6.6 mm Hg post-aspiration, with a paired mean reduction of 7.0±5.3 mm Hg (*P*<0.001). Among patients with preaspiration mean PAP of >25 mm Hg, the paired mean reduction was 8.4±5.2 mm Hg. Baseline systolic PAP also decreased significantly from 48.5±11.0 to 36.5±10.2 mm Hg, with a paired mean reduction of 12.0±9.1 mm Hg (*P*<0.001). Overall, the median intensive care unit stay was 0.3 (IQR, 0.0–1.2) days. Among the 52.3% (57/109) patients who were admitted to the intensive care unit postprocedure, the median intensive care unit stay was 1.2 (IQR, 0.9–2.2) days.

### Primary Safety End Point

**Figure 3.**
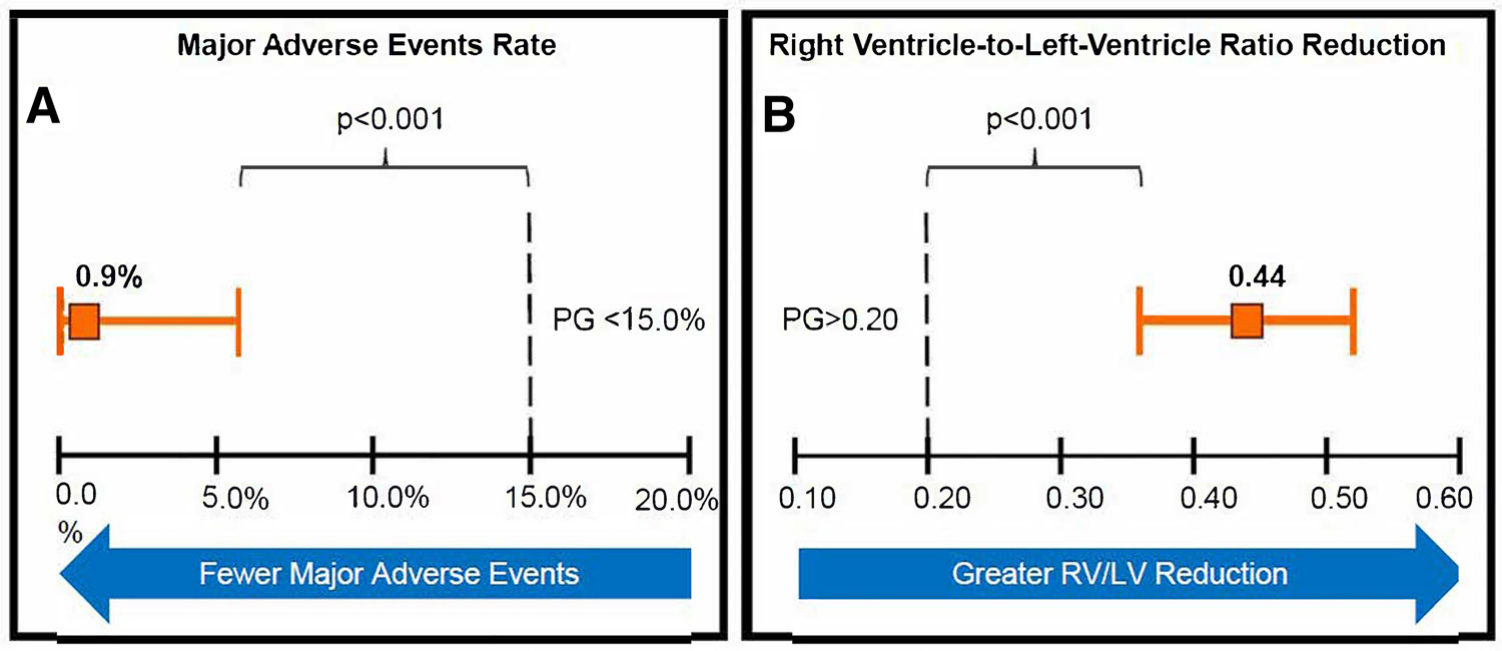
**Primary safety and efficacy end points. A**, Incidence of major adverse events within 48 hours, defined as a composite of all-cause major bleeding, device-related mortality, and serious device-related events, adjudicated by an independent academic safety board. **B**, Core laboratory–assessed reduction in right ventricle-to-left ventricle (RV/LV) ratio from baseline to 48-hour follow-up in relation to prespecified performance goals (PGs).

The independent safety board–adjudicated MAE rate in the ITT cohort was 0.9% (1/109), with a 97.5% upper CI bound of 5.7%, significantly lower than the <15.0% performance goal (*P*<0.001; Figure 3). The single MAE was a major bleeding event adjudicated based on a >3.0-g/dL decrease in hemoglobin that occurred approximately 2 days after the index procedure. The subject that experienced this event initially presented with a saddle PE that extended bilaterally into the segmental arteries, concomitant DVT, and an eligibility deviation for intracardiac thrombus that was not appreciated on initial review of baseline imaging. The index PE was successfully treated with the study device and an immediate reduction in mean PAP from 40 to 26 mm Hg; however, the subject deteriorated a few hours post-procedure, and repeat CTPA demonstrated a new PE. Repeat thrombectomy was successfully performed using the study device, and an inferior vena cava filter was placed. Following the second thrombectomy, the subject developed pulmonary reperfusion injury, and a groin hematoma was noted ≈2 days after the procedure. While the hematoma appeared stable, 1 unit of whole blood was transfused as it was unclear how much blood loss there was in the soft tissue. The subject made a full recovery and was discharged to their home ≈11 days after the index procedure.

### Primary Efficacy End Point

The core laboratory–adjudicated mean reduction in computed tomography angiogram assessed RV/LV from baseline to 48 hours post-procedure was 0.44±0.42, with a lower 97.5% CI bound of 0.36, exceeding the prespecified performance goal of >0.20 (*P*<0.001; Figure 3) in the modified intention-to-treat cohort. Data were missing for only a small proportion of subjects (3.8%; 4/106). Prespecified mean imputation and tipping point analyses confirmed that the missing data did not significantly impact study results or conclusions, with lower confidence bounds ranging from 0.32 to 0.36, all exceeding the prespecified performance goal by a wide margin (*P*<0.001).

### Prespecified Secondary Safety End Points

There was no device-related mortality, device-related clinical deterioration, device-related pulmonary vascular injury, or device-related cardiac injury at 48 hours or within the 30-day follow-up. Symptomatic PE recurrence within 30 days was reported in 2.8% (3/106) of patients. All subjects with recurrent PE presented with concomitant DVT, with DVT clearly identified as a likely source of the recurrent PE in 2 subjects. No PE-related or all-cause mortality was observed during the trial (Table 2). At least 1 serious adverse event was experienced by 11% (12/109) of subjects. The types and frequencies of serious adverse events and nonserious adverse events are summarized in Tables S7 and S8, respectively.

**Table 2.**
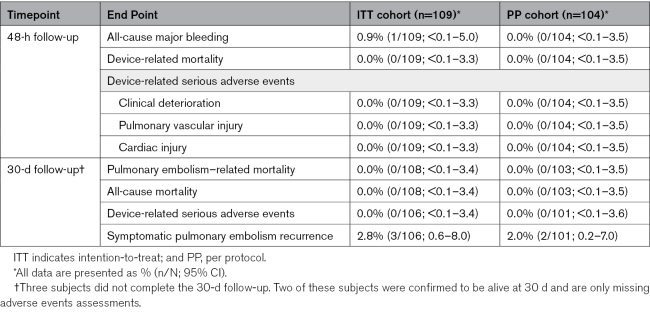
Secondary Safety End Point Results

### Prespecified Exploratory End Points

**Figure 4.**
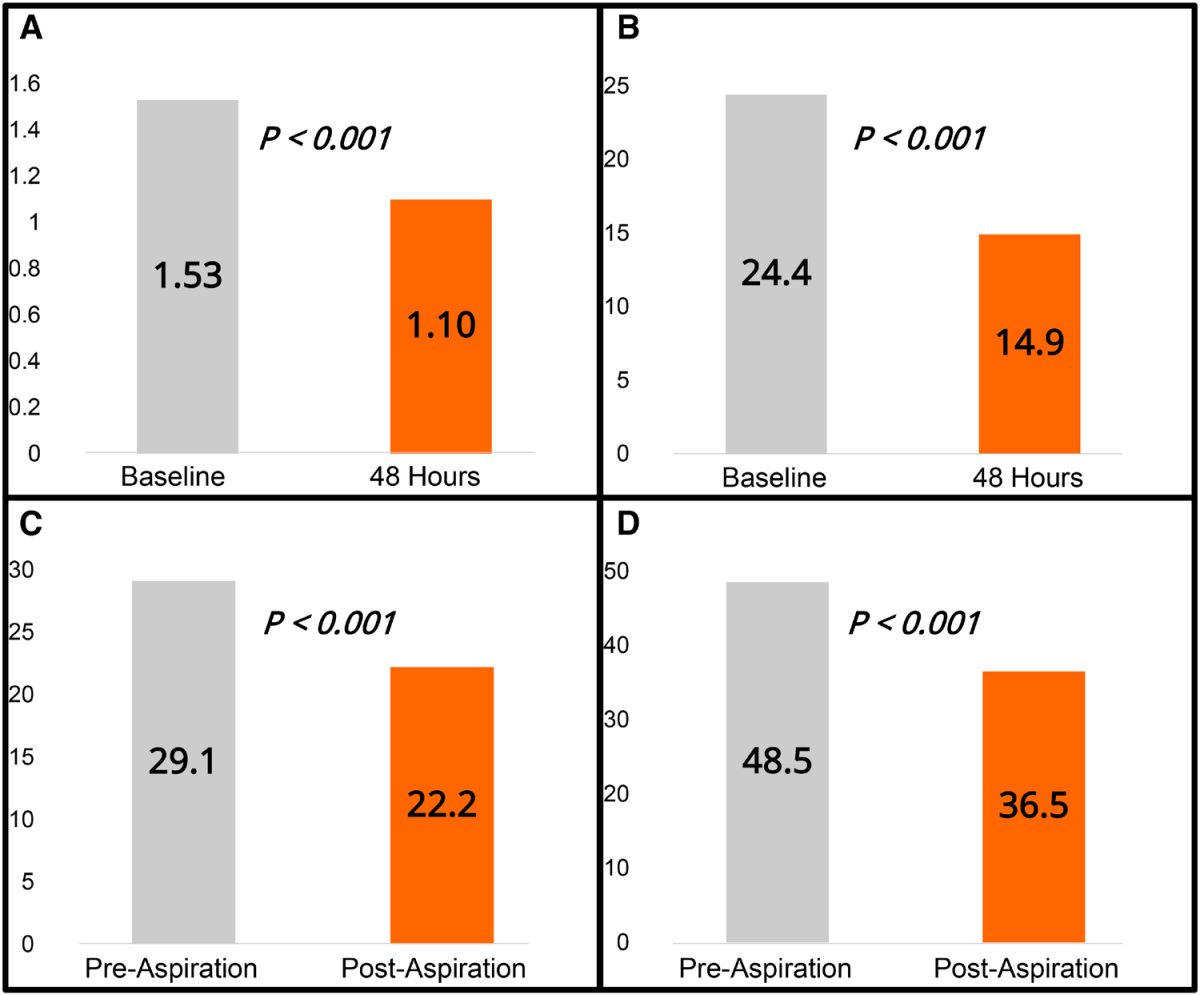
**Right ventricle-to-left ventricle (RV/LV) ratio and exploratory end point. A**, Paired core laboratory assessed reduction in the RV/LV ratio from baseline to 48-hour follow-up. **B**, Core laboratory–assessed reduction in refined modified Miller index from baseline to 48-hour follow-up. **C**, Immediate postprocedure reduction in mean pulmonary artery pressure. **D**, Immediate postprocedure reduction in systolic pulmonary artery pressure.

Core laboratory–adjudicated refined modified Miller index decreased significantly from 24.4±4.1 at baseline to 14.9±5.4 at 48 hours, with a paired mean reduction of 9.3±5.1 (*P*<0.001) that represents a 38% reduction in clot burden (Figure 4). Median aspirated blood volume, as estimated by measuring the amount of fluid (blood+saline) in the canister, was 250 (IQR, 175–350) mL. No transfusions were required during the procedure. Thrombolytics or nonstudy devices were used in 2.8% (3/109) of patients within 48-hour follow-up.

### Prespecified Subgroup Analysis Based on Catheter Size

A subgroup analysis based on the catheter size used during the thrombectomy procedure was performed with subjects divided into 1 of 2 prespecified subgroups: cases where a 24F catheter was used (N=101) and cases where a 16F catheter was used (N=44). The 24F and 16F catheters were frequently used together (N=36), with 8 procedures completed using only the 16F catheter, so the analyzed subgroups are not mutually exclusive. The primary safety and efficacy end point performance goals were met in both of the subgroup analyses, and no notable differences were observed between the subgroups. Due to the large amount of overlap between subgroups, an additional post hoc analysis was completed comparing the 65 procedures where only a 24F catheter was used to the 44 procedures where a 16F catheter was used alone or with a 24F catheter. The mean reduction in RV/LV was not significantly different (*P*=0.724) between these groups with 0.45±0.41 (95% CI, 0.35–0.56) for the 24F group and 0.42±0.43 (95% CI, 0.28–0.56) for the 16F group. The MAE rate was also not significantly different (*P*>0.999) between these groups with a 1.5% (1/65 [95% CI, 0.1–9.4]) rate in the 24F group and a 0.0% (0/44 [95% CI, 0.0–10]) rate in the 16F group.

## Discussion

The SYMPHONY IDE (Symphony Investigational Device Exemption) trial reports the safety and efficacy of the Symphony Thrombectomy System in the treatment of patients with acute intermediate-risk PE. The study met the prespecified performance goals for both the primary safety (MAE) and the primary efficacy (reduction in RV/LV) end points. Importantly, there were also meaningful reductions in PAP and modified Miller index, and a 0% mortality rate through 30 days, attesting to the efficacy and safety of this device. Reported results and data integrity were maintained through independent core laboratory adjudication and oversight by an academic independent safety board. Based on data generated in this trial, the U.S. Food and Drug Administration (FDA) granted 510(k) clearance for the use of this device in the peripheral vasculature and for the treatment of pulmonary embolism.

Patients with acute intermediate-risk PE have a significant likelihood of death (3%–15%) with anticoagulation alone. The common cause of mortality in such patients is obstructive shock, leading to progressive RV dysfunction and an RV shock spiral.^22,23^ Moreover, 1 in 3 patients with intermediate-risk PE are in normotensive shock, a state of low cardiac index despite preserved systolic blood pressure.^24^ Observational studies have shown a benefit of rapid removal of thrombus with large-bore mechanical thrombectomy on in-hospital and long-term morbidity and mortality in such patients.^25,26^ Ongoing randomized trials including PE-TRACT (The Pulmonary Embolism: Thrombus Removal with Catheter-Directed Therapy) (NCT05591118), HI-PEITHO (The Higher-Risk Pulmonary Embolism Thrombolysis) (NCT04790370), STORM-PE (A prospective, multicenter, randomized controlled trial evaluating anticoagulation alone vs anticoagulation plus computer assisted vacuum thrombectomy for the treatment of intermediate-high-risk acute pulmonary embolism) (NCT05684796), PEERLESS (A Randomized Controlled Trial of Large-Bore Thrombectomy Versus Anticoagulation in Intermediate-Risk Pulmonary Embolism) II (NCT06055920), and PERSEVERE (Randomized Controled Trial of High-Risk Pulmonary Embolism Comparing FlowTriever System vs. Standard of Care) (NCT06588634) will provide more definitive evidence for the role of mechanical thrombectomy over anticoagulation alone in such patients.

The Symphony Thrombectomy System is a novel next-generation continuous aspiration system that, by leveraging deep vacuum-pulse technology, combines the efficacy advantages of continuous mechanical aspiration (via a generator) and large-bore catheters (16F and 24F) with the safety of real-time vacuum control via the integrated handle. The ProHelix device assists in the retrieval of thrombus corked to the tip of the catheter and minimizes the likelihood of embolization. The median catheter time in the current study was 31 minutes, which is considerably faster than that of the FlowTriever device in PEERLESS (47.9 minutes)^27^ or the AVENTUS^28^ device (39.5 minutes).

For the primary safety end point, the Symphony Thrombectomy System met the prespecified performance goal with a 48-hour MAE of 0.9% (1 patient). The single MAE was a major bleed in a patient with concomitant DVT and intracardiac thrombus who required a second thrombectomy within 3 hours of the index procedure and developed a large groin hematoma 2 days post-procedure. This rate of MAE is among the lowest reported in recent pivotal thrombectomy and catheter-directed thrombolysis studies^6,19,20,28–30^ (Figure S1). In the per protocol cohort, which excludes this subject, the MAE rate was 0.0% (0/104). Moreover, there were no device-related SAEs (clinical deterioration, pulmonary vascular injury, or cardiac injury), PE-related mortality, or all-cause mortality at 30 days, further attesting to the safety of the device. The current iteration of the device does not have a blood return system, and as such, the median estimated blood loss was 250 mL based on the total fluid volume in the canister, which includes saline. While the median estimated blood loss is higher compared with thrombectomy systems with a blood return system, no patients needed blood transfusions perioperatively.

**Figure 5.**
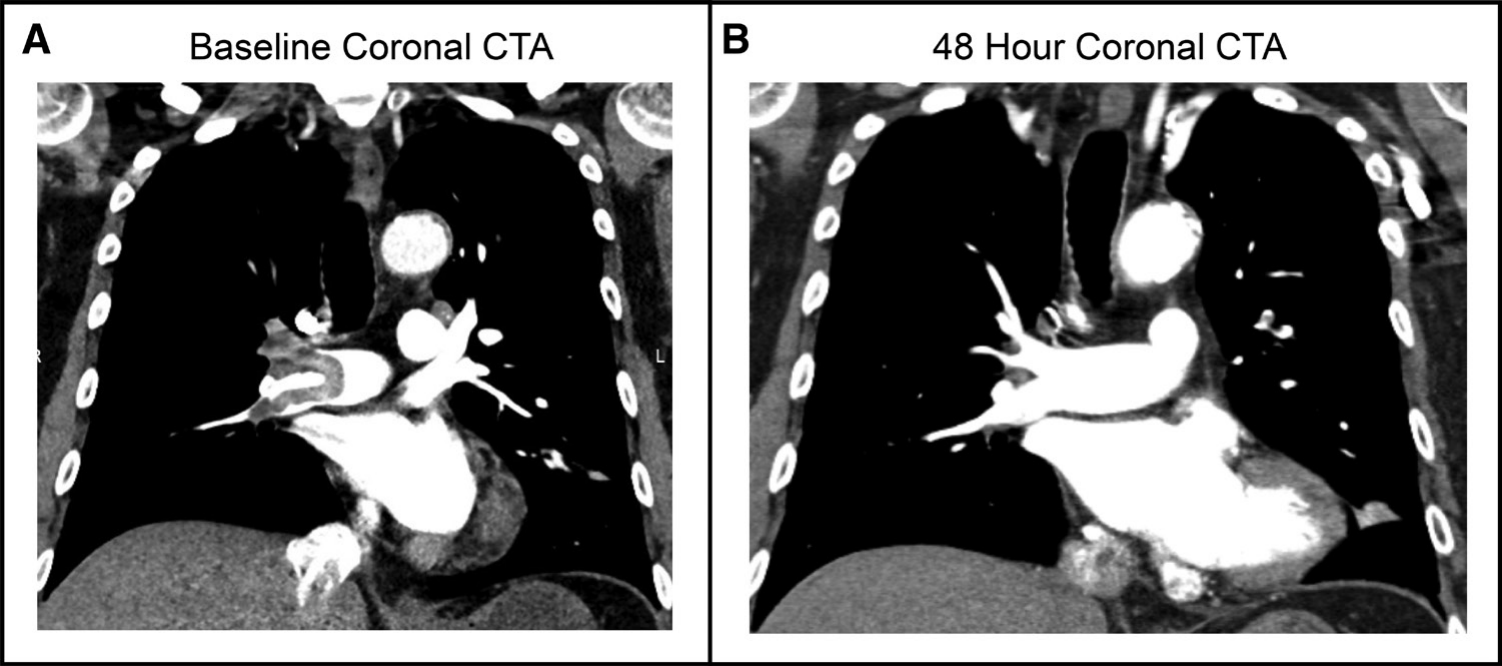
**Case summary for a subject with right pulmonary artery embolism. A**, Subject with predominantly right-sided pulmonary embolism graded as a refined modified Miller index of 19 at baseline. The subject was treated using a 24F Symphony catheter with an estimated aspirated blood volume of 400 mL and a substantial post-aspiration decrease in systolic pulmonary artery pressure (24-mm Hg reduction). **B**, Follow-up imaging demonstrated an 87% reduction in clot burden at 48 hours with a refined modified Miller index of 2.5 and a 46% decrease in right ventricle-to-left ventricle ratio from 2.03 to 1.09. The subject was discharged home later that day and experienced no adverse events through the 30-day follow-up. CTA indicates computed tomography angiogram.

**Figure 6.**
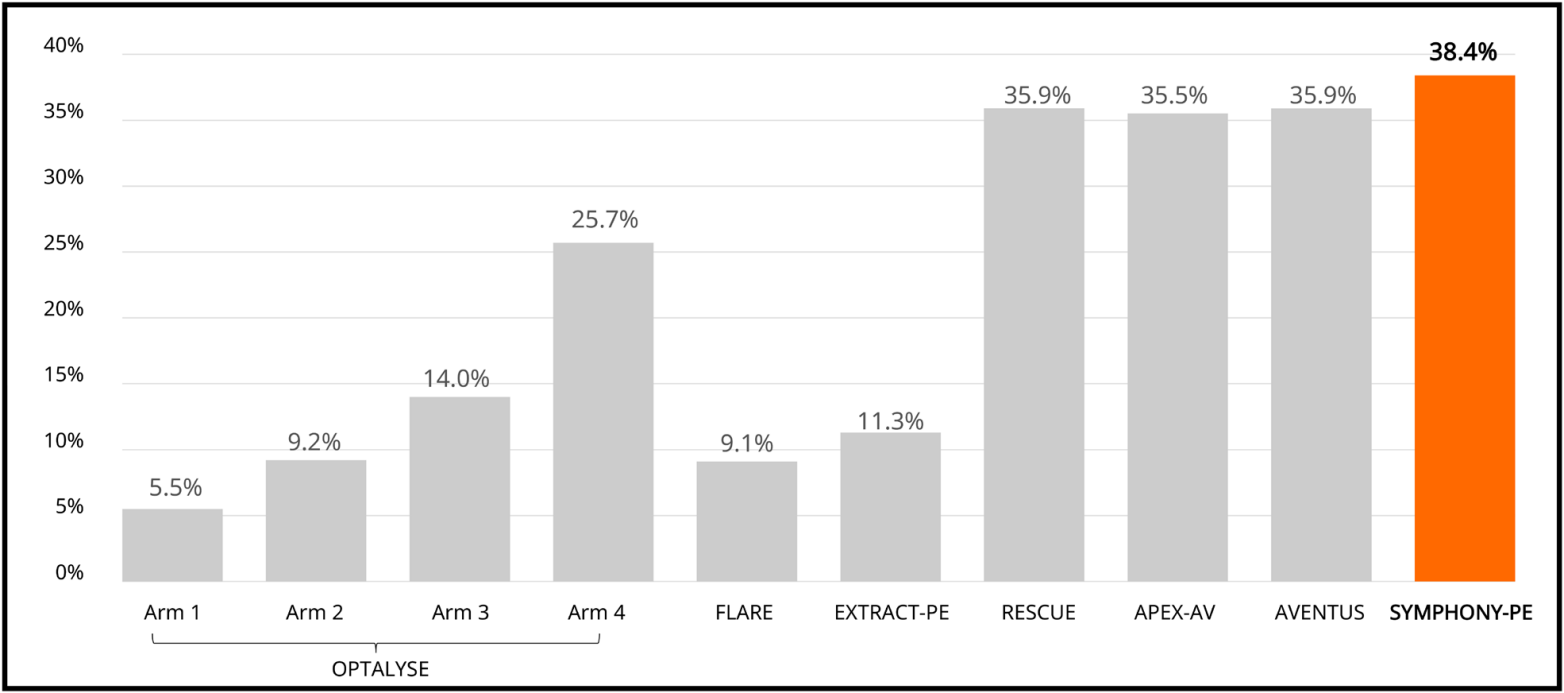
**Clot burden reduction in contemporary pulmonary embolism thrombectomy and catheter-directed thrombolysis studies.** Clot burden reduction as assessed using the refined modified Miller index (OPTALYSE, FLARE, RESCUE, APEX-AV, AVENTUS, and SYMPHONY-PE) or computed tomography obstruction index (EXTRACT-PE). For the OPTALYSE trial, all arms used the EKOS device with varied thrombolytic doses delivered (arm 1: 4 mg per lung over 2 hours; arm 2: 4 mg per lung over 4 hours; arm 3: 6 mg per lung over 6 hours; and arm 4: 12 mg per lung over 6 hours). APEX-AV indicates Acute Pulmonary Embolism Extraction with the AlphaVac system; EKOS, EkoSonic Endovascular System; EXTRACT-PE, A Prospective, Multicenter Trial to Evaluate the Safety and Efficacy of the Indigo Aspiration System in Acute Pulmonary Embolism; FLARE, FlowTriever Pulmonary Embolectomy Clinical Study; OPTALYSE; A Randomized Trial of the Optimum Duration of Acoustic Pulse Thrombolysis Procedure in Acute Intermediate-Risk Pulmonary Embolism, RESCUE, Recombinant tPA by Endovascular Administration for the Treatment of Submassive PE Using CDT for the Reduction of Thrombus Burden; and SYMPHONY-PE, Symphony Pulmonary Embolism.

For the primary efficacy end point, the mean reduction in the RV/LV ratio was 0.44 at 48 hours, and the lower confidence bound exceeded the >0.20 performance goal. This is comparable to the 0.35 to 0.56 range seen in prior pivotal thrombectomy and catheter-directed thrombolysis studies^6,19,20,28–30^ (Figure S2; Tables S9 and S10), which had similar baseline characteristics to this study (Tables S11 and S12). Furthermore, there was an immediate and significant reduction in mean and systolic PAPs post-thrombectomy. Moreover, the modified Miller index, which is an index of clot burden in the pulmonary arteries, was significantly reduced in the trial after thrombectomy, as illustrated in the case summary and computed tomography angiogram images provided in Figure 5. The 38.4% clot burden reduction in this study exceeded the clot burden reductions seen in the pivotal studies for thrombectomy devices and recent catheter-directed thrombolysis studies (Figure 6) including OPTALYSE (A Randomized Trial of the Optimum Duration of Acoustic Pulse Thrombolysis Procedure in Acute Intermediate-Risk Pulmonary Embolism)^6^ (EkoSonic Endovascular System, 5.5%–25.7%), FLARE^19^ (FlowTriever, 9.1%), EXTRACT-PE^20^ (Indigo System, 11.3%), RESCUE (Recombinant tPA by Endovascular Administration for the Treatment of Submassive PE Using CDT for the Reduction of Thrombus Burden)^29^ (BASHIR, 35.9%), APEX-AV (Acute Pulmonary Embolism Extraction with the AlphaVac system)^30^ (AlphaVac F18, 35.5%), and AVENTUS^28^ (Aventus, 35.9%).

As with other IDE trials, this study was not randomized, and as such, the results cannot be directly extrapolated for comparison with anticoagulation alone or with alternative thrombectomy devices. While the findings provide important insights into the safety and effectiveness of the mechanical thrombectomy with the study device, randomized controlled trials are desirable to further define the role of mechanical thrombectomy in treating patients with PE. Moreover, we evaluated short-term outcomes (30 days) only. Future studies, including planned registries and randomized controlled trials, will be important to assess the sustained impact of mechanical thrombectomy on clinically relevant end points such as post-PE syndrome, chronic thromboembolic pulmonary hypertension, quality of life, and other long-term outcomes. Finally, the study enrolled patients with intermediate-risk PE; thus, the results cannot be extrapolated to patients with high-risk PE or the subset of patients excluded from the trial (eg, those with clot-in-transit or severe pulmonary hypertension).

## Conclusions

This study supports the safety and effectiveness of the Symphony Thrombectomy System in the treatment of patients with acute intermediate-risk PE. There was a significant reduction in RV/LV, PAP, and clot burden with no device-related SAE and only 1 MAE associated with major bleeding. The Symphony Thrombectomy System provides a novel, efficient device that combines the efficacy of continuous mechanical aspiration using large-bore devices with the safety of real-time vacuum control.

## Article Information

### Acknowledgments

The authors thank members of the Independent Safety Board (Drs Cindy Grines, Constantino Pena, and Andrew Sharp) and the NAMSA Imaging Core Laboratory for their contributions.

### Author Contributions

All authors critically revised the manuscript.

### Sources of Funding

This study was funded by Imperative Care, Inc. There was no funding for writing the manuscript or analyzing study data.

### Disclosures

Dr Bangalore reports consulting and speaking for Abbott Vascular, Boston Scientific, Inari, Truvic/Imperative Care, Recor, and Shockwave. Dr Tomalty reports services other than consulting for Inari Medical and Imperative Care. Dr Kado is a consultant for Argon and Inari Medical. Dr Sayfo is a consultant for Terumo, Surmodics, AngioDynamics, Shockwave Medical, Medtronic, and Imperative Care. Dr Raskin reports consulting for Abbott Medical, Abiomed, Imperative Care, and Inari Medical. Dr Qamar reports consulting and speaking for Chiesi and Penumbra. Kirema Garcia-Reyes reports consulting fees from Boston Scientific, Johnson & Johnson Medical Device, Cook Medical, Teleflex, and AstraZeneca. Dr Lipshutz is a consultant for Balt USA and Inari Medical. Dr Butty is a consultant for Inari, AngioDynamics, and Imperative Care. Dr Gandhi is a consultant for Imperative Care, Endologix, Johnson and Johnson, and Cook. Dr Dexter is a consultant for AngioDynamics, Boston Scientific, Inari Medical, and Penumbra. Dr Trivax is a faculty or a speaker for Janssen Pharmaceuticals and Boston Scientific. Dr Bishay reports consulting for Imperative Care (unpaid) and Penumbra, and received consulting fees from Boston Scientific. The other authors report no conflicts.

### Supplemental Material

Tables S1–S12

Figures S1–S2

## Supplementary Material


