# Supplementary material for: Prospective Multicenter IDE Study of the Next-Generation Precision Aspiration Thrombectomy System for Intermediate-Risk Pulmonary Embolism: The SYMPHONY-PE Trial
Source: Circ Cardiovasc Interv. 2025 Sep 17;18(11):e015815. doi: 10.1161/CIRCINTERVENTIONS.125.015815 (PMC12622275; doi:10.1161/CIRCINTERVENTIONS.125.015815)
Supplement: Supplementary file 1 [file hcv-18-e015815-s001.docx]

**SUPPLEMENTAL MATERIAL**

A Prospective Multicenter IDE Study of the Next Generation Precision Aspiration Thrombectomy System for Intermediate-Risk Pulmonary Embolism: The SYMPHONY-PE Trial

**Supplemental Figures**

[**Figure S1 –** Major Adverse Event Rates in Contemporary PE Thrombectomy and Catheter Directed Thrombolysis Studies 3](#_Toc205990840)

[**Figure S2 –** RV/LV Reduction in Contemporary PE Thrombectomy and Catheter Directed Thrombolysis Studies 4](#_Toc205990841)

**Supplemental Tables**

[**Table S1 –** Study Inclusion and Exclusion Criteria 5](#_Toc205990842)

[**Table S2 –** Visit and Assessment Schedule 7](#_Toc205990843)

[**Table S3 –** Study Definitions of Major Adverse Events 8](#_Toc205990844)

[**Table S4 –** Thrombus Location and Morphology in Study Patients 10](#_Toc205990845)

[**Table S5 –** Baseline Lab Test Results 11](#_Toc205990846)

[**Table S6 –** Procedure and Device Use Characteristics 12](#_Toc205990847)

[**Table S7 –** Summary of All Serious Adverse Events 13](#_Toc205990848)

[**Table S8 –** Summary of All Non-Serious Adverse Events 14](#_Toc205990849)

[**Table S9 –** Efficacy, Safety, and Exploratory Endpoint Comparisons to Thrombectomy 510(k) Clearance Studies 15](#_Toc205990850)

[**Table S10 –** Efficacy, Safety, and Exploratory Endpoint Comparisons to Contemporary Catheter Directed Thrombolytic Studies for Acute Intermediate Risk Pulmonary Embolism 16](#_Toc205990851)

[**Table S11 –** Baseline Demographics, Clinical, and Procedural Characteristic Comparisons to Thrombectomy 510(k) Clearance Studies 17](#_Toc205990852)

[**Table S12 –** Baseline Demographics, Clinical, and Procedural Characteristic Comparisons to Contemporary Catheter Directed Thrombolytic Studies for Acute Intermediate Risk Pulmonary Embolism 18](#_Toc205990853)


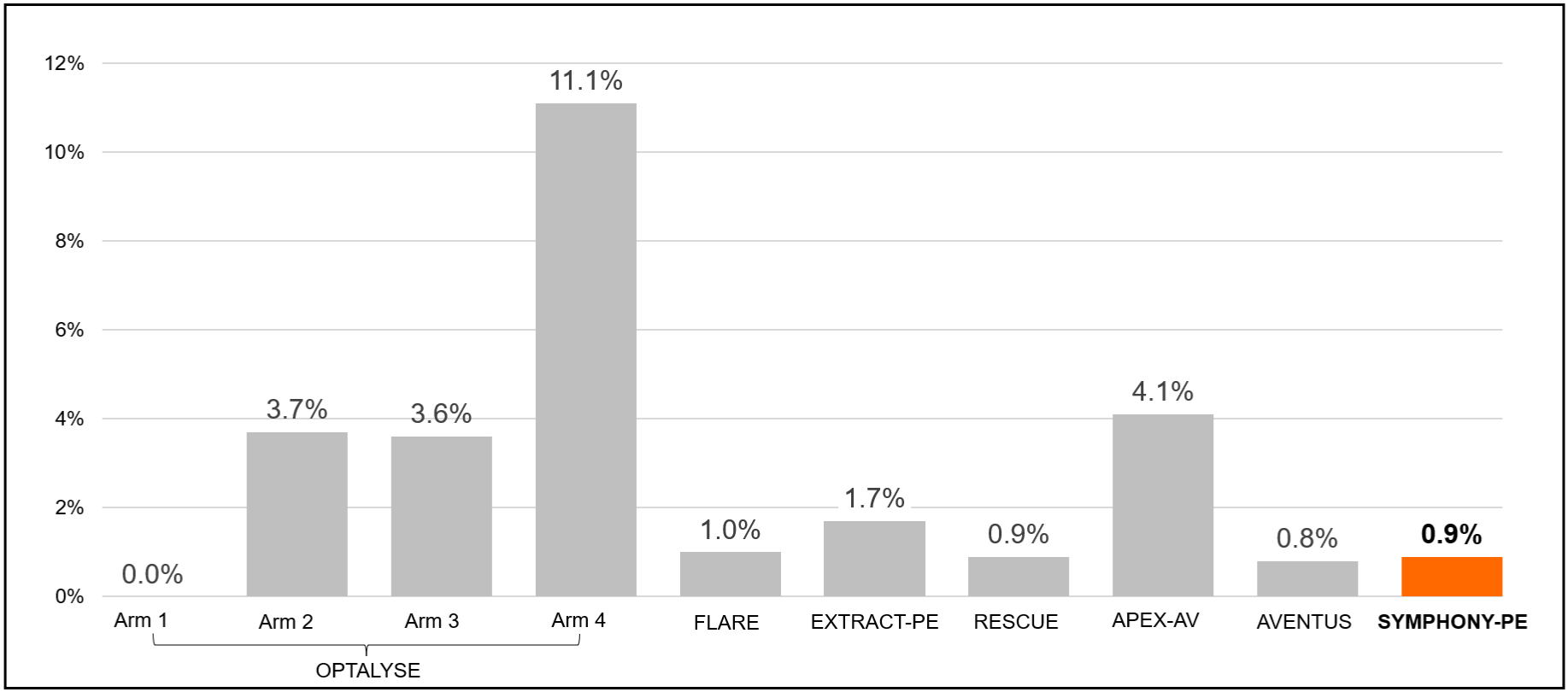


**Figure S1.** **Major adverse event (MAE) rates in contemporary pulmonary embolism thrombectomy and catheter directed thrombolysis studies.** Three (3) subjects with non-device related serious adverse events were excluded from the 3.8% (4/104) composite major adverse events rate reported in the FLARE study to better match the major adverse events definition used for SYMPHONY-PE trial and the other included studies. For the OPTALYSE trial, all arms used the EKOS device with varied thrombolytic doses delivered (Arm 1 - 4mg per lung over 2 hours, Arm 2 - 4mg per lung over 4 hours, Arm 3 - 6mg per lung over 6 hours, and Arm 4 – 12 mg per lung over 6 hours).

**
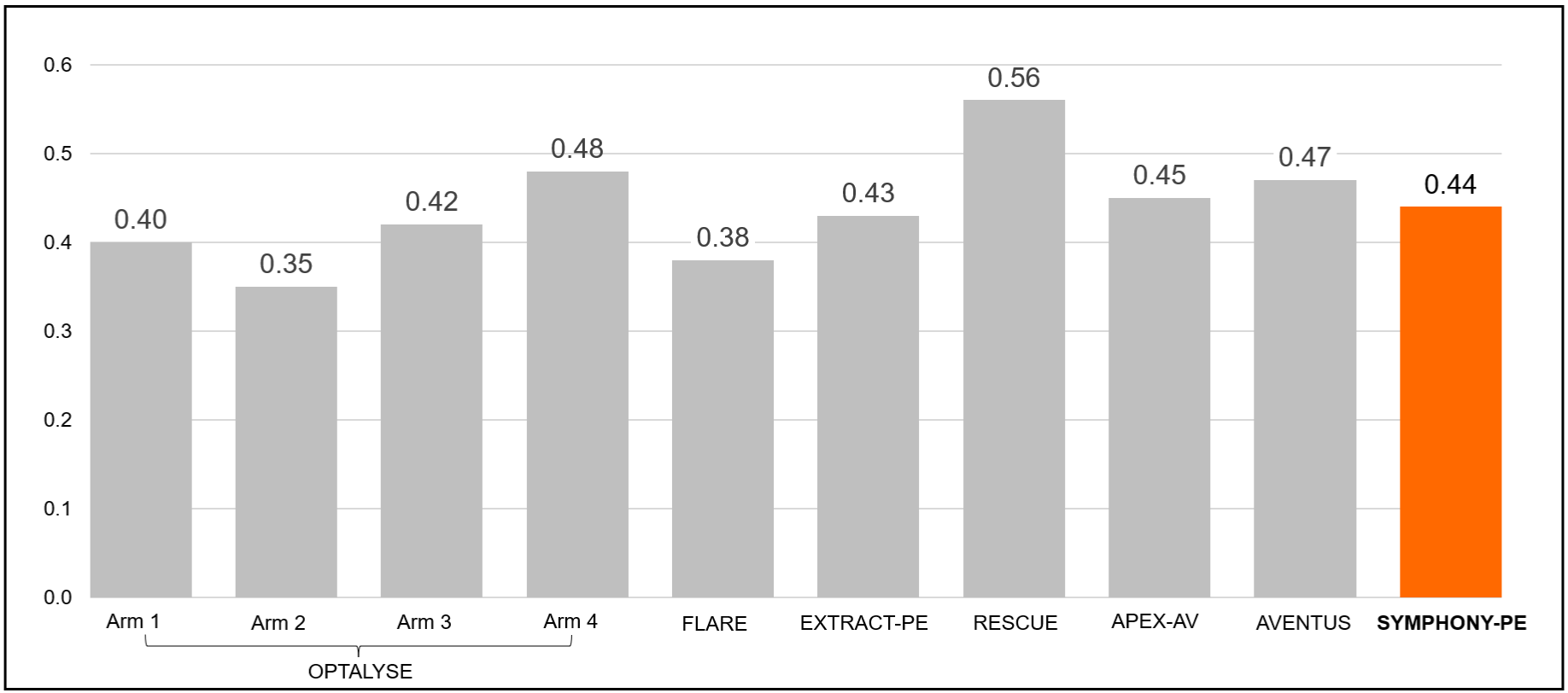
**

**Figure S2. Right ventricle to-left ventricle ratio (RV/LV) reduction in contemporary pulmonary embolism thrombectomy and catheter directed thrombolysis studies.** Right ventricle to-left ventricle ratio (RV/LV) reduction was assessed at 48 hours post intervention in all studies. For the OPTALYSE trial, all arms used the EKOS device with varied thrombolytic doses delivered (Arm 1 - 4mg per lung over 2 hours, Arm 2 - 4mg per lung over 4 hours, Arm 3 - 6mg per lung over 6 hours, and Arm 4 – 12 mg per lung over 6 hours).

**Table S1:** Study Inclusion and Exclusion Criteria

| **Inclusion Criteria** | Subjects must meet ALL of the following criteria to be eligible for participation in the study:   1. CTA evidence of acute PE within ≤14 days 2. Clinical signs and symptoms consistent with acute PE. 3. Systolic BP ≥90 mmHg with evidence of dilated RV with an RV/LV ratio >0.9 (based on Investigator’s assessment of RV/LV ratio) 4. Stable heart rate <130 BPM prior to procedure 5. Subject is between 18 and 80 years of age 6. Subject is willing to sign an IRB-approved informed consent form 7. Subject is willing and able to comply with protocol follow-up |
| --- | --- |
| **Exclusion Criteria** | 1. Thrombolytic use within 14 days of baseline CTA 2. International Normalized Ratio (INR) >3 3. Platelets <100,000/µL 4. Kidney dysfunction as confirmed by serum creatinine >1.8 mg/dL or GFR <45 mL/min 5. Hematocrit <28% or hemoglobin <9 g/dL 6. Systolic BP <90 mmHg for 15 min or requirement of inotropic support to maintain systolic BP ≥90 mmHg any time after admission 7. Experienced cardiac arrest 8. Has left bundle branch block 9. Known bleeding diathesis or coagulation disorder 10. Presence of intracardiac lead in the right ventricle or right atrium 11. Presence of intracardiac thrombus 12. Major trauma within the past 14 days 13. Cardiovascular or pulmonary surgery within last 7 days 14. Known serious, uncontrolled sensitivity to radiographic agents 15. Contraindication to anticoagulants, i.e., heparin or alternative 16. Patient on extracorporeal membrane oxygenation (ECMO) 17. Cancer requiring active chemotherapy 18. Heparin-induced thrombocytopenia (HIT) 19. Pulmonary hypertension with peak pulmonary artery pressure >70 mmHg by right heart catheterization. 20. History of chronic severe pulmonary hypertension, and/or chronic left heart disease with left ventricular ejection fraction ≤30% 21. Life expectancy <90 days as determined by investigator 22. Pregnant or nursing 23. COVID-19 positive at hospital admission 24. Current participation in another investigational study 25. Evidence such as imaging or other that suggests the subject is not appropriate for this procedure (e.g., target vessel size is too small to accommodate 16F or 24F catheters). |

**Table S2:** Visit and Assessment Schedule

| **Visits** | **Number** | **1** | **2** | **3** | **4** | **5** |
| --- | --- | --- | --- | --- | --- | --- |
|  | **Name** | **Baseline** | **Procedure** | **48-Hour**  **Follow-Up**  **(-8/+24 Hrs)** | **Discharge** | **30-Day Follow-Up**  **(± 7 Days)** |
| **Assessments** | | | | | | |
| **Informed Consent** | | X |  |  |  |  |
| **Demographics and Medical History** | | X |  |  |  |  |
| **Eligibility Criteria** | | X | X |  |  |  |
| **Vitals** | | X | X | X | X | X |
| **Labs** | | X | X | ^*^ | ^*^ | ^*^ |
| **CT Angiography** | | X | X | X |  |  |
| **Procedure Metrics** | |  | X |  |  |  |
| **Concomitant Medications** | | X | X | X | X | X |
| **Adverse Events Assessment** | |  | X | X | X | X |
| **Study Exit** | |  | ^*^ | ^*^ | ^*^ | X |

^*^ Completed at any time as necessary

**Table S3:** Study Definitions of Major Adverse Events

| **Term** | **Definition** |
| --- | --- |
| **Major Adverse Events** | Major Adverse Event (MAE) was defined as a composite of:   1. Major bleeding within 48 hours 2. Device-related mortality within 48 hours 3. Device-related SAEs within 48 hours, including:    - Clinical deterioration    - Pulmonary vascular injury    - Cardiac injury |
| **Major Bleeding** | Major Bleeding was defined as any life threatening, disabling, or major bleed as assessed per the Valve Academic Research Consortium-2 (VARC-2) bleeding definitions. This definition was selected for use in this study to match the definitions used in prior studies for other mechanical thrombectomy devices.  VARC-2 Life Threating or Disabling Bleed  Any of the following types of bleeds:   - Fatal bleeding - Bleeding in a critical organ, such as intracranial, intraspinal, intraocular, or pericardial necessitating pericardiocentesis, or intramuscular with compartment syndrome - Bleeding causing hypovolemic shock or severe hypotension requiring vasopressors or surgery - Overt source of bleeding with drop in hemoglobin ≥ 5 g/dL or whole blood or packed red blood cells (RBCs) transfusion of ≥ 4 units   VARC-2 Major Bleeding  Overt bleeding that is associated with any of the following:   - Associated with a drop in the hemoglobin level of at least 3 g/dL - Requires transfusion of 3 units of whole blood/RBC^*^ - Causes hospitalization, permanent injury, or requiring surgery   VARC-2 Minor Bleeding  Any bleeding worthy of clinical mention (e.g., access site hematoma) that does not qualify as life-threatening, disabling, or major bleeding.  *Note: According to the VARC-2 Consensus Document it is critical to acknowledge that a bleeding complication has to be the result of overt bleeding and cannot be adjudicated based on blood transfusion alone. Some blood loss through the lumen of an aspiration catheters is an expected result of a thrombectomy procedure and necessary when removing large clot burdens. |
| **Clinical Deterioration** | Clinical deterioration was defined as any of the following conditions:   - Unexpected requirement for intubation or mechanical ventilation - Arterial hypotension (>1 hour or requiring vasopressors) or shock - Cardiopulmonary resuscitation - Persistent worsening in oxygenation - Emergency surgery |
| **Pulmonary Vascular Injury** | Pulmonary vascular injury was defined as perforation or injury of a major pulmonary arterial branch during the index procedure and may include arterial venous fistula, dissection, hemorrhage, perforation, rupture or thromboembolic occlusion resulting in permanent damage. |
| **Cardiac Injury** | Cardiac injury was defined as any damage to the heart during the index procedure, including but not limited to an event necessitating blood transfusion, acute myocardial infarction, arrhythmia requiring intervention, cardiac hematoma, or tricuspid or pulmonic valve damage. |
| **Mortality** | Mortality was defined as the occurrence of a death at any point during or following the index procedure. |
| **Symptomatic PE Recurrence** | Symptomatic PE recurrence was assessed in the subset of subjects that showed a clear clinical improvement following treatment with the study device. Symptomatic PE recurrence was defined as a worsening in PE symptoms between baseline and post procedure, with evidence of new or worsened thrombus burden confirmed via CTA. PE symptoms include, but are not limited to chest pain, dyspnea, hemoptysis, palpitations, and tachycardia. |

**Table S4:** Thrombus Location and Morphology in Study Patients

| **Characteristic^*^** | **IIT Cohort (N=109)**  **Rate, % (n/N)** | **mITT Cohort (N=106)**  **Rate, % (n/N)** |
| --- | --- | --- |
| **PE Thrombus Location** |  |  |
| Saddle Only | 11% (12/109) | 11% (12/106) |
| Bilateral Only | 52% (57/109) | 53% (56/106) |
| Saddle + Bilateral | 31% (34/109) | 31% (33/106) |
| Saddle + Unilateral | 0.9% (1/109) | 0.0% (0/106) |
| Unilateral Only, Left Side | 0.0% (0/109) | 0.0% (0/106) |
| Unilateral Only, Right Side | 4.6% (5/109) | 4.7% (5/106) |
| **Right Thrombus Locations** | 89% (97/109) | 89% (94/106) |
| Right Main | 82% (89/109) | 81% (86/106) |
| Right Lobar | 66% (72/109) | 66% (70/106) |
| Right Segmental | 58% (63/109) | 59% (62/106) |
| **Left Thrombus Locations** | 83% (91/109) | 84% (89/106) |
| Left Main | 68% (74/109) | 68% (72/106) |
| Left Lobar | 61% (66/109) | 60% (64/106) |
| Left Segmental | 56% (61/109) | 56% (59/106) |
| **Thrombus Morphology** |  |  |
| Acute | 61% (67/109) | 62% (66/106) |
| Sub-acute | 57% (62/109) | 58% (61/106) |
| Chronic | 16% (17/109) | 15% (16/106) |

^*^ Thrombus location and thrombus morphology categories are not mutually exclusive, and totals may exceed 100%.

**Table S5:** Baseline Laboratory Test Results

| **Lab Tests** | **ITT Cohort (N=109)^*^** | |
| --- | --- | --- |
|  | **Mean ± SD**  **(Minimum – Maximum)** | **Median**  **[IQR]** |
| Hemoglobin, g/dL | 13.9 ± 1.7 (9.9 - 19) | 13.8  [12.7 - 15.1] |
| Hematocrit, % | 42 ± 4.9 (29 - 56) | 42  [39 - 45] |
| Platelet Count, thousands/µL | 213 ± 72  (105 - 502) | 195  [164 - 239] |
| INR | 1.1 ± 0.2 (0.9 - 1.8) | 1.1  [1.0 - 1.2] |
| Serum Creatinine, mg/dL | 1.0 ± 0.2 (0.6 - 1.6) | 1.0  [0.8 - 1.2] |
| eGFR, mL/min | 77 ± 18 (47 - 119) | 75  [63 - 92] |
| Troponin I, ng/L  (new high sensitivity test) | 259 ± 349 (2 - 1536) | 127 [51 - 261] |
| Troponin T, ng/L (new high sensitivity test) | 105 ± 140 (11 - 793) | 62  [28 - 128] |
| Lactate, mmol/L | 1.9 ± 1.3 (0.5 - 8.1) | 1.7  [1.3 - 2.0] |
| BNP, pg/mL | 440 ± 823 (10 - 4058) | 125  [61 - 368] |
| NT-proBNP, pg/mL | 1844 ± 2149 (36 - 9039) | 933  [346 - 2544] |
| D-Dimer, ng/mL FEU | 13280 ± 22633 (944 - 150000) | 8770  [5327 - 14185] |

^*^ Sample sizes varied based on lab test.

**Table S6:** Procedure and Device Use Characteristics

| **Characteristic** | **IIT Cohort (N=109)^*^** | **mITT Cohort (N=106)^*^** |
| --- | --- | --- |
| **Sedation** |  |  |
| Conscious | 89% (97/109) | 89% (94/106) |
| General | 2.8% (3/109) | 2.8% (3/106) |
| None | 8.3% (9/109) | 8.5% (9/106) |
| Used 24F Symphony only | 60% (65/109) | 60% (64/106) |
| Used 24F and 16F Symphony | 33% (36/109) | 32% (34/106) |
| Used 16F Symphony only | 7.3% (8/109) | 7.5% (8/106) |
| Used ProHelix | 11% (12/109) | 11% (12/106) |
| Symphony Use Time, minutes, median [IQR] | 31 [20 – 53] | 31 [20 – 52] |
| Total Procedure Time, minutes, median [IQR] | 61 [41 – 85] | 61 [41 – 85] |

Unless indicated otherwise, data are presented as % (n/N)

**Table S7:** Summary of All Serious Adverse Events

| **Serious Adverse Event Term** | **ITT Cohort (N=109)**  **Rate, % (n/N)** |
| --- | --- |
| Pulmonary Embolism (recurrent or residual)7F | 3.7 (4/109) |
| Respiratory failure | 1.8 (2/109) |
| Hemorrhage or excessive blood loss | 1.8 (2/109) |
| Pulmonary Injury^†^ | 0.9 (1/109) |
| Thrombosis | 0.9 (1/109) |
| Pneumonia | 0.9 (1/109) |
| Arrhythmia, including ventricular fibrillation | 0.9 (1/109) |
| Infection (access site, respiratory tract, etc.) | 0.9 (1/109) |
| Other – Fall secondary to osteoarthritis exacerbation and weakness | 0.9 (1/109) |
| Other – Urinary tract infection | 0.9 (1/109) |
| Other – Obstructive renal stone | 0.9 (1/109) |

Three subjects with concomitant deep vein thrombosis experienced symptomatic pulmonary embolism recurrence. The etiology for the fourth pulmonary embolism was unable to be determined.

^†^The subject that experienced this event initially presented with a saddle pulmonary embolism that extended bilaterally into segmental arteries, concomitant deep vein thrombosis, and an eligibility deviation for intracardiac thrombus that was not appreciated on initial review of baseline imaging. The index pulmonary embolism was successfully treated with the study device. The subject deteriorated a few hours post-procedure and repeat imaging demonstrated a new pulmonary embolism. Repeat thrombectomy was successfully performed using the study device and an inferior vena cava filter was placed. Following the second thrombectomy, the subject developed pulmonary reperfusion injury, and a groin hematoma was noted approximately 2 days after the procedure. The subject made a full recovery and was discharged to their home approximately 11 days after the index procedure.

**Table S8:** Summary of All Non-Serious Adverse Events

| **Serious Adverse Event Term** | **ITT Cohort (N=109)**  **Rate, % (n/N)** |
| --- | --- |
| Hemorrhage or excessive blood loss | 2.8 (3/109) |
| Thrombocytopenia | 2.8 (3/109) |
| Anemia | 1.8 (2/109) |
| Angina | 1.8 (2/109) |
| Hematoma | 1.8 (2/109) |
| Hypotension | 1.8 (2/109) |
| Arrhythmia, including ventricular fibrillation | 0.9 (1/109) |
| Hemoptysis | 0.9 (1/109) |
| Pleural effusion | 0.9 (1/109) |
| Rise in serum creatinine levels | 0.9 (1/109) |
| Leukocytosis | 1.8 (2/109) |
| Back pain | 0.9 (1/109) |
| Chest pain | 0.9 (1/109) |
| Groin pain | 0.9 (1/109) |
| Left shoulder pain | 0.9 (1/109) |
| Acute cystitis with hematuria | 0.9 (1/109) |
| Cardiomyopathy | 0.9 (1/109) |
| Cervical disc herniation | 0.9 (1/109) |
| Clot fever | 0.9 (1/109) |
| Diastolic heart failure | 0.9 (1/109) |
| Elevated liver Function Tests | 0.9 (1/109) |
| Hyperglycemia | 0.9 (1/109) |
| Hypocalcemia | 0.9 (1/109) |
| Shortness of breath | 0.9 (1/109) |
| Transient hypotension | 0.9 (1/109) |
| Transient hypoxia | 0.9 (1/109) |
| Urinary retention | 0.9 (1/109) |
| Worsening of bilateral pleural effusions | 0.9 (1/109) |

**Table S9 –** Efficacy, Safety, and Exploratory Endpoint Comparisons to Thrombectomy 510(k) Clearance Studies

| **Endpoint Comparison** | **FLARE** | **EXTRACT-PE** | **APEX-AV** | **AVENTUS** | **SYMPHONY-PE** |
| --- | --- | --- | --- | --- | --- |
| Post-Procedure Mean PAP Reduction, mmHg, Average ± SD, % Reduction^*^ | 2.0  (6.7%) | N/A | 6.1  (21.9%) | 4.8 ± 5.5  (16.8%) | 7.0 ± 5.2  (24.0%) |
| Estimated Aspirated Blood Volume (Blood + Saline), mL range, % | N/A | 0-400: 73%  >400: 27% | 0-500: 94%  >500: 5.7% | N/A^†^ | 0-400: 84%  400-500: 12%  >500: 3.8% |
| 48-Hour Thrombolytic or Non-Study Device Use, % (n/N) | 1.9 (2/106) | 5.9 (7/119) | 2.5 (3/122) | 0.8 (1/120) | 2.8 (3/109) |
| 48-Hour Percent Reduction in Clot Burden^‡^, Average | 9.1% | 11.3% | 35.5% | 35.9% | 38.4% |
| 48-Hour Reduction in RV/LV, Average ± SD | 0.38 | 0.43 ± 0.26 | 0.45 ± 0.27 | 0.47 ± 0.36 | 0.44 ± 0.42 |
| 48-Hour Composite MAE Rate,  % (n/N) | 1.0 (1/104)^§^ | 1.7 (2/119) | 4.1 (5/122) | 0.8 (1/120) | 0.9 (1/109) |
| 30-Day All-Cause Mortality,  % (n/N) | 1.0 (1/104) | 0.8 (1/119) | 0.0 (0/122) | 0.9 (1/113)^**^ | 0.0 (0/108) |
| 30-Day Symptomatic PE Recurrence, % (n/N) | 1.9 (2/104)^††^ | 0.0 (0/119) | 0.8 (0/118) | 0.9 (1/113) | 2.8 (3/106) |

^*^ Non-paired calculation for all studies based on average baseline PAP.

^†^ Only reported net blood loss after autologous blood return which was not used in any of the other completed studies.

^‡^ Assessed using the Modified Miller Index (SYMPHONY-PE, FLARE, APEX-AV, and AVENTUS) or CT obstruction index (EXTRACT-PE)

^§^ Three (3) subjects with non-device related SAEs were excluded from the 3.8% (4/104) composite MAE rate reported in the FLARE publication to better match the MAE definition used for SYMPHONY-PE and the other IDE studies.

^**^ All-cause mortality was not reported for AVENTUS; however, there was at least one death which was described in an SAE summary.

^††^ Reported as pulmonary embolism under serious adverse events in FLARE publication.

**Table S10:** Efficacy, Safety, and Exploratory Endpoint Comparisons to Contemporary Catheter Directed Thrombolytic Studies for Acute Intermediate Risk Pulmonary Embolism

| **Endpoint Comparison** | **OPTALYSE** | | | | **RESCUE** | **SYMPHONY-PE** |
| --- | --- | --- | --- | --- | --- | --- |
|  | Arm 1 | Arm 2 | Arm 3 | Arm 4 |  |  |
| Post-Procedure Systolic PAP Reduction, mmHg, Average ± SD, % Reduction^*^ | N/A | N/A | N/A | N/A | 5.9 ± 11  (12.0%) | 12.0 ± 9.1  (24.0%) |
| 48-Hour Thrombolytic or Non-Study Device Use^†^, % (n/N) | 3.6 (1/28) | 3.7 (1/27) | 3.6 (1/28) | 0.0 (0/18) | N/A | 2.8 (3/109) |
| 48-Hour Percent Reduction in Clot Burden^‡^, Average | 5.5% | 9.2% | 14.0% | 25.7% | 35.9% | 38.4% |
| 48-Hour Reduction in RV/LV, Average ± SD | 0.40 ± 0.37 | 0.35 ± 0.27 | 0.42 ± 0.32 | 0.48 ± 0.51 | 0.56 ± 0.41 | 0.44 ± 0.42 |
| 48-Hour Composite MAE Rate, % (n/N) | 0.0 (0/27) | 3.7 (1/27) | 3.6 (1/28) | 11.1 (2/18) | 0.9 (1/109) | 0.9 (1/109) |
| 30-Day All-Cause Mortality,  % (n/N) | 0.0 (0/27) | 0.0 (0/27) | 0.0 (0/28) | 5.6 (1/18) | 0.9 (1/104) | 0.0 (0/108) |
| 30-Day Symptomatic PE Recurrence, % (n/N) | 0.0 (0/27) | 0.0 (0/27) | 3.6 (0/28) | 0.0 (0/18) | 0.0 (0/104) | 2.8 (3/106) |

^*^ Non-paired calculation for all studies based on average baseline PAP.

^†^ Rates for OPTALYSE calculated based on counts in Figure 1 of the primary publication.

^‡^ Assessed using the refined Modified Miller Index for all listed studies.

**Table S11:** Baseline Demographics, Clinical, and Procedural Characteristic Comparisons to Thrombectomy 510(k) Clearance Studies

| **Characteristic** | **FLARE** | **EXTRACT-PE** | **APEX-AV** | **AVENTUS** | **SYMPHONY-PE** |
| --- | --- | --- | --- | --- | --- |
| Female, % (n/N) | 46 (48/104) | 45 (53/119) | 56 (68/122) | 44 (53/120) | 39 (43/109) |
| Age, Average ± SD | 56 ± 14 | 59 ± 15 | 62 ± 15 | 59 ± 12 | 59 ± 13 |
| BMI, Average ± SD | 36 ± 9 | Women: 37 ±10  Men: 32 ± 7 | N/A | 36 ± 7 | 35 ± 8 |
| **Race, % (n/N)** |  |  |  |  |  |
| Asian | 0.0 (0/104) | 2.5 (3/119) | N/A | N/A | 1.8 (2/109) |
| Black or African American | 16 (17/104) | 31 (37/119) | 28 (34/122) | 17 (20/116) | 29 (32/109) |
| White | 83 (86/104) | 61 (72/119) | 69 (84/122) | 80 (93/116) | 65 (71/109) |
| Other | 0.9 (1/104) | 5.9 (7/119) | 3.3 (4/122) | 2.6 (3/116) | 3.7 (4/109) |
| Prior pulmonary embolism (PE),  % (n/N) | 9.6 (10/104) | 18 (21/119) | N/A | 8.3 (10/120) | 16 (17/109) |
| Prior or Concomitant DVT,  % (n/N) | 70 (73/104) | 61 (72/119) | N/A | 84 (86/102) | 63 (69/109) |
| Baseline RV/LV,  Average ± SD | 1.56 ± 0.34 | 1.47 ± 0.30 | 1.51 ± 0.31 | 1.57 ± 0.40 | 1.53 ± 0.44 |
| Baseline Modified Miller Index,  Average ± SD | 20.8 ± 2.4 | N/A | 15.2 ± 1.6 | 24.0 ± 4.6 | 24.4 ± 4.1 |
| Baseline Mean PAP,  Average ± SD, mmHg | 29.8 | N/A | 27.8 ± 7.8 | 28.5 ± 7.8 | 29.1 ± 7.2 |
| Device Use Time,  Median [IQR], Minutes | N/A^*^ | 37  [24 – 60] | N/A^*^ | 39.5 | 31  [20 – 53] |

^*^ FLARE only reported mean device use time (57 minutes) and APEX-AV only reported mean device/procedure time (37 minutes).

**Table S12:** Baseline Demographics, Clinical, and Procedural Characteristic Comparisons to Contemporary Catheter Directed Thrombolytic Studies for Acute Intermediate Risk Pulmonary Embolism

| **Characteristic** | **OPTALYSE** | | | | **RESCUE** | **SYMPHONY-PE** |
| --- | --- | --- | --- | --- | --- | --- |
|  | Arm 1 | Arm 2 | Arm 3 | Arm 4 |  |  |
| Female, % (n/N) | 43 (12/28) | 44 (12/27) | 61 (17/28) | 39 (7/18) | 39 (42/109) | 39 (43/109) |
| Age, Average ± SD | 59 | 62 | 60 | 59 | 57 ± 13 | 59 ± 13 |
| BMI, Average ± SD | 36 ± 11 | 36 ± 11 | 40 ± 9 | 29 ± 7 | 33 ± 6 | 35 ± 8 |
| **Race, % (n/N)** |  |  |  |  |  |  |
| Asian | 0.0 (0/28) | 3.7 (1/27) | 0.0 (0/28) | 0.0 (0/18) | N/A | 1.8 (2/109) |
| Black or African American | 43 (12/28) | 37 (10/27) | 32 (9/28) | 61 (11/18) | 29 (32/109) | 29 (32/109) |
| White | 57 (16/28) | 52 (14/27) | 68 (19/28) | 61 (11/18) | 59 (64/109) | 65 (71/109) |
| Other | 0.0 (0/28) | 7.4 (2/27) | 0.0 (0/28) | 5.6 (1/18) | 12 (13/109) | 3.7 (4/109) |
| Prior pulmonary embolism (PE), % (n/N) | 25 (7/28) | 15 (4/27) | 21 (6/28) | 11 (2/18) | 14 (15/109) | 16 (17/109) |
| Prior or Concomitant DVT,  % (n/N) | 39 (11/28) | 37 (10/27) | 50 (14/28) | 61 (11/18) | 26 (28/109) | 63 (69/109) |
| Baseline RV/LV,  Average ± SD | 1.47 ± 0.39 | 1.43 ± 0.33 | 1.49 ± 0.37 | 1.51 ± 0.58 | 1.66 ± 0.41 | 1.53 ± 0.44 |
| Baseline Modified Miller Index, Average ± SD | 20.3 ± 3.0 | 19.7 ± 4.6 | 21.1 ± 2.4 | 20.4 ± 4.1 | 22.4 ± 3.9 | 24.4 ± 4.1 |
| Baseline Systolic PAP,  Average ± SD, mmHg | N/A | N/A | N/A | N/A | 49.5 ± 13.4 | 48.5 ± 11.0 |
| Device Use Time,  Median [IQR], Minutes | N/A | N/A | N/A | N/A | N/A^*^ | 31  [20 – 53] |

^*^ RESCUE only reported mean procedure time (54 minutes).
